# Unveiling the mycobiome of healthy and Esca diseased grapevines: fungal community dynamics across different microhabitats, seasons, years, and cultivars

**DOI:** 10.1186/s40793-026-00911-w

**Published:** 2026-06-12

**Authors:** Adrienn Geiger, Carla Mota Leal, Zoltán Karácsony, Richárd Golen, Luca Annamária Lepres, Anna Molnár, Kálmán Zoltán Váczy, József Geml

**Affiliations:** 1 HUN-REN-EKKE Lendület Environmental Microbiome Research Group, Eszterházy Károly Catholic University, Eger, 3300 Hungary; 2 Food and Wine Research Centre, Eszterházy Károly Catholic University, Eger, 3300 Hungary; 3 Doctoral School of Environmental Sciences, Hungarian University of Agriculture and Life Sciences, Gödöllő, 2100 Hungary

**Keywords:** Grapevine trunk diseases (GTDs), Microhabitat, Vintage, Plant pathology, Soil ecology, Mycobiome

## Abstract

**Background:**

Grapevines host diverse microbial communities, including fungal pathogens associated with grapevine trunk diseases (GTDs), which pose a major challenge in viticulture. Despite extensive research, the ecological drivers shaping fungal community composition across different grapevine microhabitats remain insufficiently understood. Using ITS2 metabarcoding, we characterized fungal communities in bark, wood, and soil from Esca symptomatic and asymptomatic vines, and evaluated the effects of season, year, cultivar, microhabitat, and plant health on key functional groups.

**Results:**

Seasonal variation had limited effects on fungal richness, whereas interannual differences had a strong impact on functional group diversity. Microhabitat played a key role: plant pathogens, particularly GTD-associated taxa, were most prominent in bark and wood; mycoparasites were dominant in bark; generalist saprotrophs prevailed in soil and bark; and GTD-related wood saprotrophs were most abundant in bark. Soil harbored the highest overall fungal diversity. Plant health status significantly affected only GTD pathogens, which showed higher abundance and diversity in symptomatic vines. Cultivar influenced only the richness of GTD-related wood saprotrophs.

**Conclusions:**

Year (vintage) emerged as a major driver of fungal community composition across plant tissues and soil, driving the most pronounced shifts in the mycobiome. Abiotic factors (year and season) were most strongly associated with soil fungal communities, whereas biotic factors (cultivar and plant health) exerted a greater influence on fungi associated with grapevine tissues. These findings underscore the central role of microhabitat and temporal variation in structuring the grapevine mycobiome.

**Supplementary Information:**

The online version contains supplementary material available at 10.1186/s40793-026-00911-w.

## Background

Fungi play a critical role in grapevine health, with various species either benefiting or harming the plant depending on their interactions. Of particular concern to growers are the fungal pathogens that cause grapevine trunk diseases (GTDs), which can lead to severe economic losses [1]. GTDs are characterized by the colonization of the perennial organs, leading to wood decay and foliar symptoms. As a result, the blockage of water-transporting vessels can cause the death of the shoots, canes, and eventually the entire plant [2, 3, 4]. The main syndromes associated with adult vines suffering from GTDs include Esca, Botryosphaeria dieback, and Eutypa dieback [5]. In particular, the incidence of Esca has been increasing across various vine-growing regions in Europe and Hungary [6, 7]. Understanding the factors influencing the development of these fungal diseases and the expression of GTD symptoms remains a topic of ongoing research and discussion [8].

Omic techniques, particularly next-generation sequencing (NGS), have become valuable tools for assessing the microbiome of grapevine, and understanding the microbial dynamics associated with GTDs. Identifying the microbial composition of grapevine is crucial not only for developing effective disease management strategies but for gaining a deeper understanding of the complex interactions between the pathogens and host plants [9]. Early NGS studies focused on characterizing the microbiome of specific grapevine parts. In one such study, the soil microbiome surrounding Esca-diseased plants was analyzed, suggesting that bulk soil could act as a reservoir for Esca-related pathogens [10]. Notably, this study detected both bacterial and fungal communities and presented the functional roles of the most abundant taxa. Another study examined the wood mycobiome of asymptomatic and Esca-symptomatic plants, revealing that the fungal composition of Esca-affected plants closely resembled that of healthy plants [11]. While these foundational studies provided valuable insights into compartment-specific community shifts, they did not comprehensively address the functional roles of the detected fungi by classifying the fungal taxa into functional groups such as saprotrophs, pathogens, or mycoparasites.

As previous studies have indicated, the plant microbiome is strongly niche specific, with different parts of the plant hosting distinct microbial communities [12, 13, 14]. This niche specificity suggests that each microhabitat within a grapevine, from the inner woody tissue to the bark and surrounding rhizosphere soil, could harbor unique microbial communities with potentially significant roles in plant health and disease progression. However, the full extent of this compartmentalization, particularly in the context of GTDs and their associated pathogens, remains underexplored. A deeper understanding of how these microbial communities are structured across different microhabitats is crucial, as it may reveal key interactions that contribute to the persistence and spread of pathogens like those involved in GTDs. By examining multiple grapevine compartments, we can better comprehend how these niche-specific microbial populations interact with both the plant host and each other, ultimately influencing disease dynamics and overall vine health.

Abiotic stressors such as soil composition and climate conditions have been linked to the manifestation of GTD symptoms. These factors suggest that environmental conditions may exacerbate disease symptoms, implying that pathogens alone are not solely responsible for symptom development [8]. Additionally, biotic factors, including grapevine cultivar, rootstock, and overall plant physiology, contribute to the spread and severity of GTDs [15, 16]. However, there is still limited information on the precise mechanisms by which abiotic and biotic factors influence GTD symptom expression in grapevines [17]. In this study, we considered both abiotic (vintage, season) and biotic (cultivar, health state) factors, in addition to microhabitat, because each of these has been implicated in shaping plant-associated microbiomes and may interact to influence GTD progression. Temporal factors such as vintage and season capture interannual climatic variability and short-term environmental fluctuations, both of which can modulate microbial community dynamics and plant stress responses [18]. Cultivar represents the genetic and physiological background of the host, which can affect susceptibility to fungal colonization, while health state reflects the outcome of host–pathogen interactions and may be associated with shifts in community composition. Here, we focused on four cultivars (leányka, kékfrankos, cabernet sauvignon, and chardonnay), all of which were planted on the same rootstock more than 40 years ago in the same vineyard. These cultivars were selected because they represent varieties of high relevance in the region, while their shared long-term establishment under identical rootstock and site conditions allows us to disentangle cultivar-specific effects on the grapevine mycobiome from other viticultural variables. Finally, microhabitat defines the immediate ecological niche, as soil, bark, and woody tissues differ in their physical, chemical, and biological properties, likely acting as strong filters that shape fungal community assembly.

To gain deeper insights into the mycobiome associated with grapevine and soil, we designed a two-year sampling scheme aimed at investigating the roles of cultivar, health state, season, and vintage in shaping the diversity and composition of fungal communities across different microhabitats (inner woody tissue, bark, and upper rhizosphere soil) of both asymptomatic and Esca-symptomatic grapevines. We used ITS2 region metabarcoding as it is the official fungal DNA barcode and the most widely adopted marker for metabarcoding studies of fungal communities facilitating direct comparability with previous grapevine mycobiome studies. ITS2 allows high taxonomic resolution at the genus level across most fungal lineages, although species-level identification is not always reliable. Therefore, we restricted taxonomic inference to the genus level, and the functional assignment was based on genera’s primary lifestyle.

We aimed not only to examine plant pathogens, with a particular focus on GTD pathogens, but to characterize other significant functional groups within the grapevine mycobiome. To provide an ecologically meaningful framework for interpreting fungal community patterns, we classified fungal taxa into six major functional groups. (i) GTD pathogens are fungi directly implicated in grapevine trunk diseases and are capable of colonizing woody tissues, causing vascular blockage and wood necrosis. (ii) Non-GTD pathogens are fungal plant pathogens that can infect grapevine but are not typically associated with trunk disease syndromes. (iii) Generalist saprotrophs represent fungi with broad substrate ranges that play a key role in decomposition and nutrient cycling; in grapevine, they may compete with pathogens for space and resources. (iv) Mycoparasites are fungi that parasitize other fungi, and may therefore act as natural antagonists of GTD pathogens, potentially mitigating disease progression. (v) GTD-related wood saprotrophs include taxa associated with wood decay processes that overlap functionally with GTD pathogens, facilitating tissue degradation and potentially predisposing vines to infection. (vi) Non-GTD wood saprotrophs are wood-decaying fungi not directly implicated in trunk diseases but which may influence microbial succession in woody tissues by altering habitat structure and resource availability.

Through this approach, we sought to address the following research questions [1]. Do asymptomatic and Esca-symptomatic grapevines differ in terms of fungal abundance and richness across various compartments [2]? How are different functional groups (such as saprotrophs, GTD-associated taxa, and mycoparasites) distributed across distinct grapevine microhabitats (inner wood, bark, and rhizosphere soil) [3]? To what extent do host- and environment-related factors (vintage, cultivar, health state, and season) influence the structure and composition of the grapevine mycobiome?

By addressing these questions, our goal was to better understand the ecological patterns shaping the grapevine-associated fungal community and its potential role in GTD symptom development and progression.

## Methods

### Field sampling

In autumn 2019, a preliminary vineyard survey was conducted to identify grapevine plants showing typical foliar symptoms of Esca disease. The experimental site was located at the vineyard of Eszterházy Károly Catholic University, near Eger, Hungary. The overall severity of Esca in this vineyard was low, with symptomatic vines representing less than 5% of the total population. From four different scion cultivars, two local (leányka and kékfrankos) and two international (chardonnay and cabernet sauvignon), two symptomatic vines per cultivar were selected and marked in 2019. Symptomatic vines were classified according to the presence of interveinal chlorosis, tiger-stripe patterns on leaves, marginal leaf necrosis, showing these symptoms on multiple shoots. Each symptomatic plant was paired with two neighboring asymptomatic vines (with no visible symptoms of Esca or other GTDs), resulting in a total of 12 marked vines per cultivar (2 symptomatic + 2 × 2 asymptomatic = 6 vines × 4 cultivars = 24 vines in total).

All selected vines were grafted on Teleki 5 C rootstock. The vineyard was established approximately 40 years ago on brown forest soil with clay eluviation and rhyolite tuff bedrock. The site experiences a continental climate typical of the Carpathian Basin hills. The vineyard has been managed under consistent cultivation practices and uniform abiotic conditions over the past four decades, with the sampled cultivars planted in close proximity. In 2020, tebufenpyrad was applied to the foliage in May to control mites, and a broad-spectrum insecticide containing lambda-cyhalothrin was sprayed in July. No pesticides were applied directly to the soil.

Sampling was conducted at four time points: February and August of 2020, and February and August of 2021. Samples were collected from the same marked vines at each time point to ensure consistency. No leaf samples were included in the study; only bark, wood, and rhizosphere soil were analyzed. While initial vine selection in 2019 was based on visible Esca foliar symptoms, no disease symptoms were recorded during subsequent sampling events, as symptom expression in GTDs is known to be intermittent, plants may exhibit symptoms in one year and appear asymptomatic the next.

Soil samples were collected from the rhizosphere by sampling four points within a 50 cm radius around the base of each vine, to a depth of 20 cm. Bark samples were peeled from four points on each trunk using a sterile scalpel, and wood samples were drilled from the same peeled areas. The drill was sterilized with 4% (w/v) available chlorine bleach between samples. All four subsamples from each material type were pooled per plant and sampling time, resulting in a composite sample for each microhabitat (bark, wood, and soil), per plant and time point. In total, 48 composite samples were collected per sampling time point (3 microhabitats × 4 cultivars × 4 plants per cultivar = 48), and immediately transported to the laboratory, where they were stored at − 80 °C.

All samples were lyophilized and homogenized using a TissueLyser LT (Qiagen, Venlo, The Netherlands) with 2 mm steel beads. Genomic DNA was extracted from approximately 0.5 ml of each homogenized composite sample using either the NucleoSpin^®^ Soil or Plant DNA extraction kit (Macherey-Nagel GmbH & Co., Düren, Germany). No extraction blank control was included, which may limit the detection of contaminants introduced during DNA extraction; however, a PCR negative control was included to monitor contamination during amplification. Fungal ITS2 region amplicons were generated using primers ITS7f [19] and ITS4 [20], with each forward and reverse primer tagged using unique 8-nucleotide barcodes for sample identification. For amplification, genomic DNA from *Candida albicans* ATCC 10,231 (American Type Culture Collection) served as a positive control, while ultra-clean, DNA-free water (Merck) was included as a negative control. Amplicon libraries were sequenced on an Illumina MiSeq platform using 250 bp paired-end reads.

### DNA metabarcoding data analysis

The *dada2* (v.3.16) R package [21] was used to process the raw DNA sequences in the R statistical computing environment. Raw sequences were truncated to 240 and 200 base pairs for forward and reverse reads, respectively, and subsequently were quality-filtered with default settings, denoised, merged, chimera filtered, and clustered into amplicon sequence variants (ASVs). Taxonomic assignments of fungal sequences were made based on the 2022, October 16 version of the UNITE reference database [22], using USEARCH v. 11 [23]. A minimum identity threshold of 97% and minimum coverage of 80% were required for assignment. Species-level assignments were only accepted above 99% identity; otherwise, taxa were retained at the genus level. Rarefaction curves were generated based on ASV count tables using the *vegan* R package [24] with random subsampling without replacement across a range of sequencing depths to assess sampling completeness and compare estimated fungal richness among samples. We used the FungalTraits reference database [25] to assign ASVs with genus-level identification to functional groups, such as plant pathogens, wood saprotrophs, generalist saprotrophs, and mycoparasites. In the functional group of generalist saprotrophs, we encompassed soil saprotrophs, unspecified saprotrophs, nectar/tap saprotrophs, dung saprotrophs, litter saprotrophs, pollen saprotrophs sooty molds. Within the functional guild of plant pathogens and wood saprotrophs, we further distinguished pathogenic genera with known association with GTDs, based on literature search. All sequences of ASVs analyzed in this paper have been submitted to GenBank (KIEY0000000) and a BioProject (PRJNA1073309) was generated. The links for the sequences and the BioProjects are in the Availability of data and materials section.

### Statistical analysis

All statistical analyses were done in R [26]. The fungal community matrix was normalized (rarefied) by random subsampling to the smallest library size (13,046 reads) on a per-sample basis. Singleton ASVs were excluded and the resulting rarefied matrix contained 3418 ASVs, that served as input for the subsequent analyses. We used ANOVA and Tukey’s HSD test to compare ASV richness and abundance of different functional groups among samples, which were graphically presented as boxplots using the *ggplot2* R package [27].

Compositional differences were visualized using non-metric multidimensional scaling (NMDS) in the *vegan* R package [27], with Bray-Curtis distance measure on the Hellinger-transformed matrices for all samples, and for samples collected from the different microhabitats. We performed PerMANOVA (adonis) in *vegan* to estimate the amount of variation explained by the tested variables, i.e., microhabitat, health state, cultivar, season, and year for all samples, and for samples collected from the different microhabitats, respectively. Homogeneity of multivariate dispersion was tested using the *betadisper* function in *vegan* to assess whether significant PERMANOVA results reflected differences in centroid location rather than group dispersion. Taxonomic information of asymptomatic and symptomatic wood samples was plotted with the package *metacoder* [28], using the *heat_tree* function. Community composition patterns were visualized by plotting the relative abundance of the fungal taxa across different microhabitats and years using *ggplot2* R package [25].

## Results

Of the 2206 ASV we identified to genus level, plant pathogenic fungi were represented by 573 ASVs, including 223 ASVs of GTD-associated plant pathogens. Mycoparasites were represented by 99 ASVs, GTD-related wood saprotrophs by 32 ASVs, non-GTD wood saprotrophs by 286 ASVs, and generalist saprotrophs by 705 ASVs. Among non-GTD plant pathogens, the most diverse genera were *Fusarium* (44 ASVs), *Alternaria* (31), and *Devriesia* (21), while dominant GTD-associated pathogens included *Diplodia* (48), *Phaeomoniella* (41) and *Eutypella* (41). Mycoparasitic fungi were largely represented by *Cystobasidium* (19), *Trichoderma* (19), and *Papiliotrema* (11), while *Lophiotrema* (18), *Peniophora* (13), and *Keissleriella* (11) were the most diverse wood decomposers, the majority of GTD-related wood saprotrophs belonged to *Kalmusia* (11) and *Stereum* (4). Regarding generalist saprotrophs, the genera *Solicoccozyma* (61), *Mortierella* (45) and *Vishniacozyma* (40) were the most diverse. Rarefaction curves approached saturation for most samples (Fig. S1), indicating that sequencing depth was generally sufficient to capture the majority of fungal diversity. Differences in asymptotic richness values among samples reflect variation in community diversity across microhabitats and experimental factors.

**Fig. 1:**
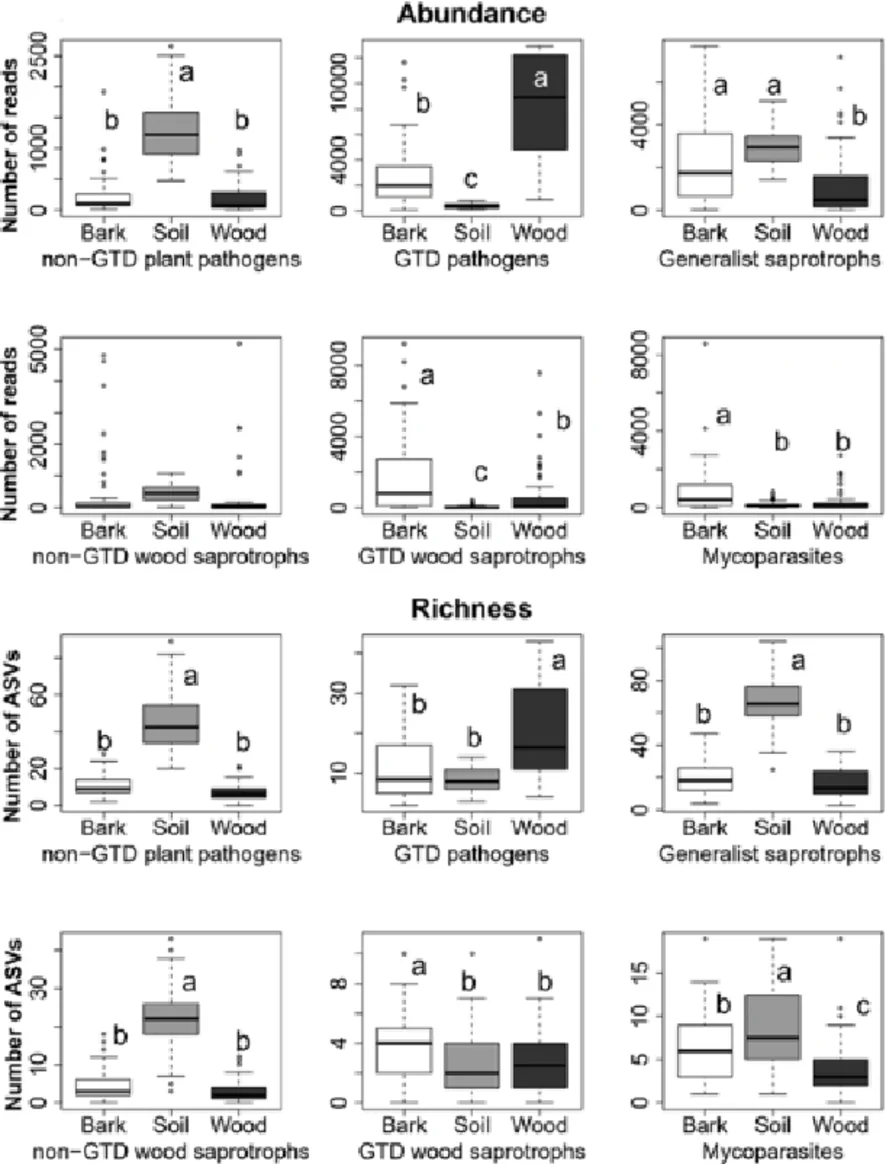
.

Non-GTD plant pathogens were abundant in soil, while GTD pathogens were abundant in wood followed by bark (Fig. 1). Generalist saprotrophs were abundant in soil and bark, non-GTD related wood saprotrophs were represented equally in the different microhabitats in terms of abundance, but with several outliers in bark and wood. (Fig. 1) GTD wood saprotrophs were abundant in bark, followed by wood, while mycoparasites were abundant mostly in bark (Fig. 1).

**Fig. 2:**
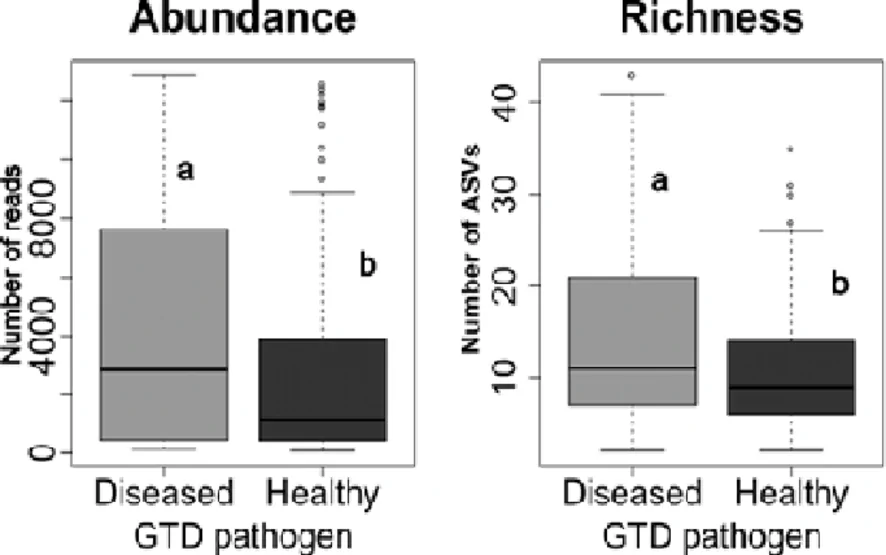
Rarefied abundance and richness of GTD pathogens in the diseased and healthy plants. Letters indicate significant differences in one-way ANOVA post hoc Tukey HSD test (*p* < 0.05)

All functional groups seemed to be unaffected by health state, abundance and richness values remained the same in asymptomatic and Esca symptomatic plants, except for GTD pathogens, the samples collected from diseased plants showed higher abundance and diversity (Fig. 2).

**Fig. 3:**
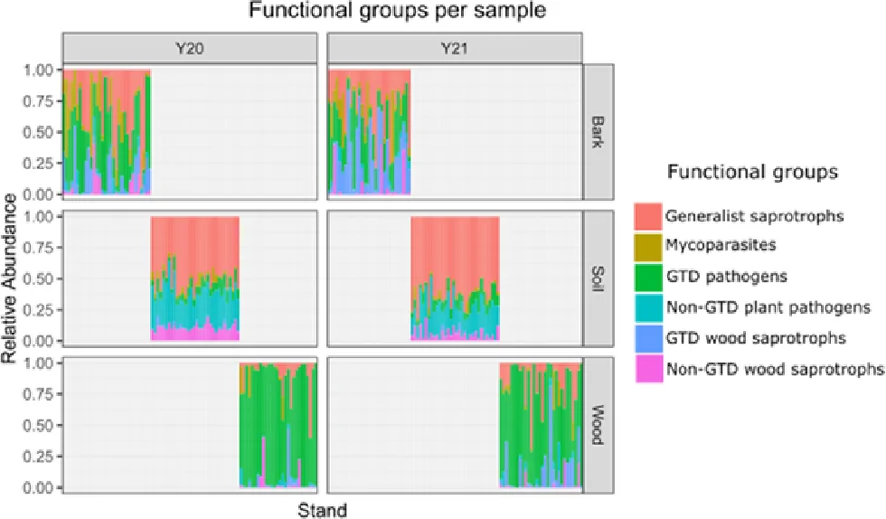
Relative abundance of the functional groups, separated by source and year. Bars represent proportional composition of functional groups within each sample, grouped by source (rows) and sampling year (columns). Colors indicate different ecological categories of fungi

The abundance of *Botryosphaeria* and *Eutypella*, and the richness of *Botryosphaeria*, *Eutypella*, and *Fomitiporia* species were significantly higher in symptomatic plants. Seasonal changes affected in the abundance of wood saprotrophs, and in the richness of the mycoparasites and GTD-related wood saprotrophs. Annual differences were more pronounced, affecting the abundance of plant pathogens and GTD-related wood saprotrophs, as well as the richness of plant pathogens, GTD pathogens, and wood saprotrophs. To visualize these patterns, we generated stacked barplots showing the relative composition of functional groups across microhabitats and vintages (Fig. 3). Soil was consistently dominated by generalist saprotrophs, wood by GTD pathogens, while bark harbored a more heterogeneous community with contributions from both mycoparasites and GTD-related wood saprotrophs. A clear interannual shift was evident in all compartments, with relative abundances declining in 2021 compared to 2020. (Fig. 3)

Abundance and richness values dropped of the affected functional groups in the year of 2021 compared to 2020. The only functional group affected by cultivar was GTD-related wood saprotrophs, being the most diverse in kékfrankos while the least diverse in chardonnay. Overall, microhabitat was the most determining factor modifying the abundance and richness of the examined functional groups.

**Fig. 4:**
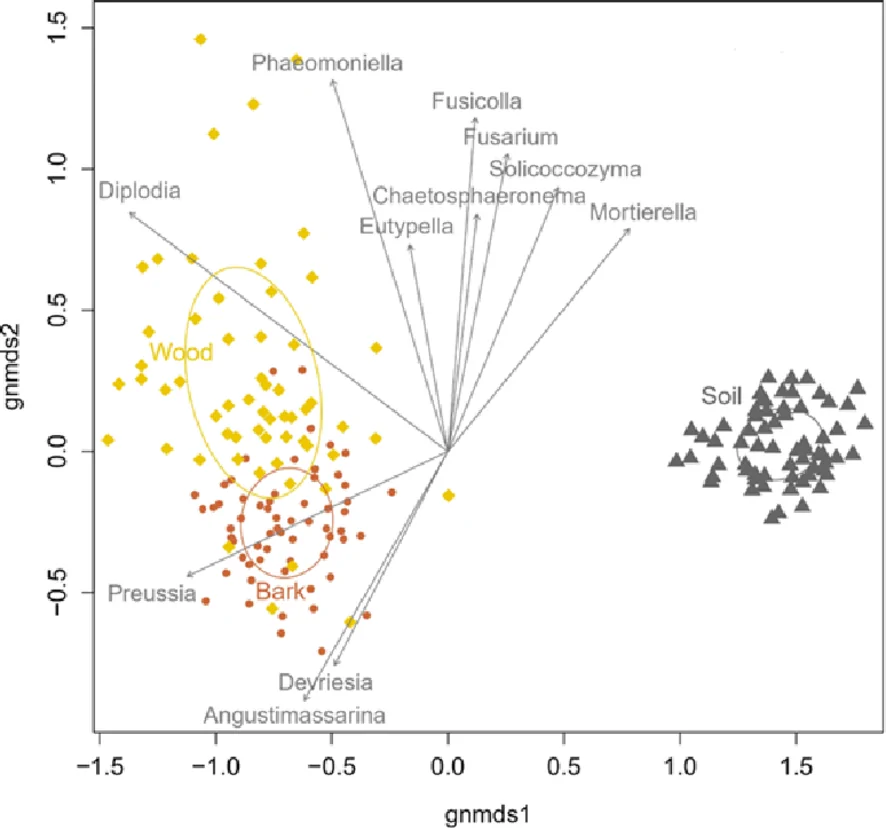
Non-metric multidimensional scaling (NMDS) ordination plot of the fungal community composition among sampling sources (stress value = 0.1195879). The ordination was based on a Bray-Curtis distance matrix generated from the abundance table. Ellipses indicate the different microhabitats, while arrows show 11 out of the 20 most diverse genera with p-values less than or equal to 0.05. Longer arrow means stronger association

The fungal community composition was visualized in a two-dimensional NMDS ordination for all the samples (stress value = 0.1195879) (Fig. 4). Based on PerMANOVA, microhabitat (*p* = 0.001), year (*p* = 0.001), cultivar (*p* = 0.019) and season (*p* = 0.039) showed significant correlation with the fungal community structure. Tests of multivariate dispersion indicated no significant differences in variance for year (*p* = 0.86), health state (*p* = 0.45), cultivar (*p* = 0.74), or season (*p* = 0.13), confirming that these PERMANOVA effects reflect genuine compositional shifts. In contrast, dispersion was significant for microhabitat (*p* < 0.001), suggesting that part of the strong microhabitat effect may be explained by unequal variability among soil, bark, and wood samples. Microhabitat explained 3.8% of the variation, while year 2.5%, cultivar 1.5%, and season 1.2% of the variation. Samples collected from soil can be clearly distinguished from samples of bark and wood, while the latter two are overlapping each other (Fig. 4). Health state did not correlate significantly with the community structure, when all ASVs were examined together. Vectors on the NMDS plot represent the most diverse genera significant with ordination axes, and thus contributing significantly to the observed patterns.

**Fig. 5:**
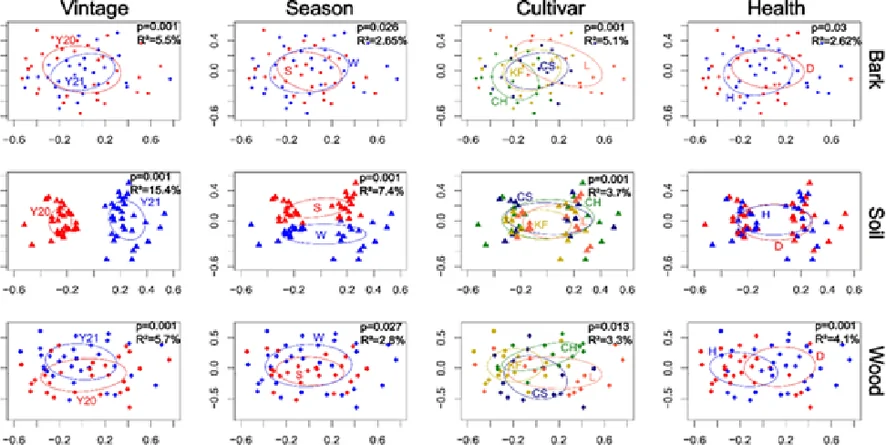
Non-metric multidimensional scaling (NMDS) ordination plots of the fungal community composition across bark (stress value = 0.2759618), wood (stress value = 0.2675174) and soil (stress value = 0.1908301). The ordination was based on a Bray-Curtis distance matrix generated from the abundance table. The plots display the same ordination across the microhabitats with ellipses showing the standard deviation of compositional differences among samples from the same year (**a**Y20-2020; Y21 -2021), season (**b** W-winter, S-summer), cultivar (**c** KF-kékfrankos, L-leányka, CH-chardonnay, CS-cabernet sauvignon) and health state (**d** H-healthy, D-Diseased)

Fungal community composition was visualized in a two-dimensional NMDS ordination separately for the different microhabitats (Fig. 5). In bark (stress value = 0.2759618), cultivar (*p* = 0.001), year (*p* = 0.001), season (*p* = 0.026), and health (*p* = 0.03) all showed significant correlation with the fungal community structure. Year explained the greatest variation (5.5%) among bark samples. Community composition of bark-associated fungi differed most between the two white varieties (chardonnay, leányka). In soil (stress value = 0.1908301), year (*p* = 0.001), cultivar (*p* = 0.001) and season (*p* = 0.001) correlated with the fungal community structure. The greatest variation (15.4%) among the soil samples was explained by interannual changes, with year (i.e., vintage) explaining more than 15% of compositional differences among soil samples. For wood (stress value = 0.2675174), all categorical variables showed significant correlation with the community structure (cultivar (*p* = 0.013), year (*p* = 0.001), season (*p* = 0.027), health (*p* = 0.001)), with again vintage explaining the highest proportion of variance (5.7%) among samples. Vintage was the most determining driver of the mycobiome composition within the microhabitats, particularly evident in the case of soil, where it caused the greatest shift.

**Fig. 6:**
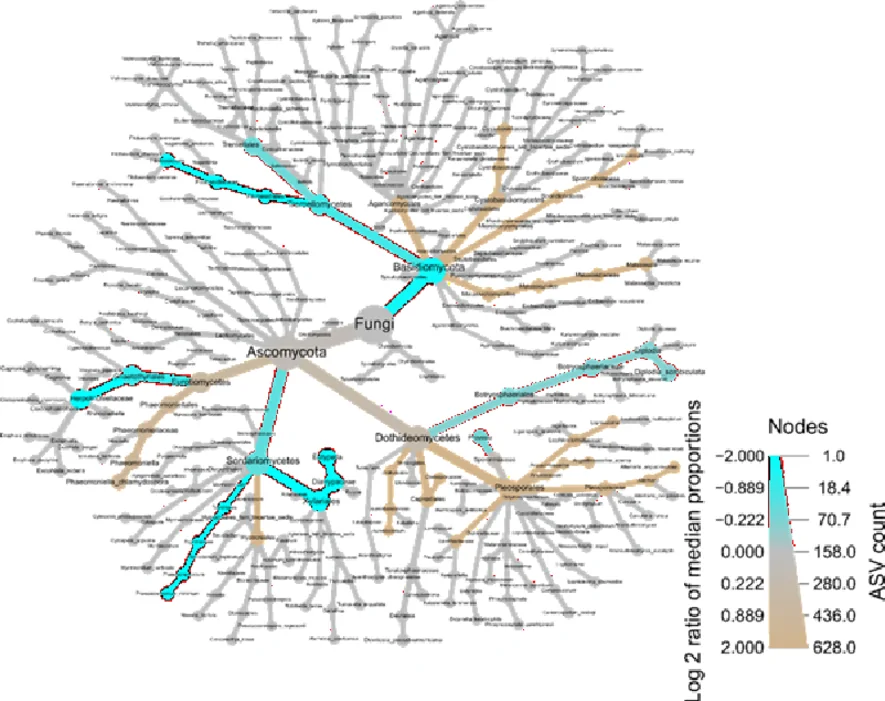
Differential heat tree showing significant differences in median read proportion between fungal ASVs of healthy (brown) and diseased (cyan) wood samples. For each taxon a Wilcoxon Rank Sum test was used to test for differences between the median abundances of samples in each treatment. Only taxa that appear in at least 5 samples are shown

As health state had noticeable impact on the variation (4.1%) of wood samples, a differential heat tree of the wood compartment was created (Fig. 6). In diseased wood, the ascomycetous GTD related genera *Diplodia* (*Botryosphaeriaceae*), *Phaeoacremonium* (*Togniniaceae*) and *Eutypella* (*Diatrypaceae*) were highly abundant, as was the basidiomycetous genus *Filobasidium* (*Tremellales*) (Fig. 6). In healthy wood, the ascomycetous GTD-related genera *Phaeomoniella* (*Phaeomoniellaceae*) were prevalent, along with *Aureobasidium*, and *Alternaria*, and some members of *Hypocreales* order (Fig. 6). Additionally, the basidiomycetous genera *Rhodotorula* and *Malassezia* were dominant in healthy wood (Fig. 6).

## Discussion

This study provides a comprehensive assessment of how microhabitat, seasonality, vintage, cultivar, and health state shape the mycobiome of grapevines. Fungal communities differed more strongly between below- and aboveground compartments than among years, seasons, cultivars, or health states. Moreover, the influence of these factors varied by microhabitat: soil fungi correlated most with abiotic drivers (year and season), whereas biotic factors (cultivar and health state) were more important in bark and wood communities that are in direct contact with the host.

Across all samples, microhabitat emerged as the strongest driver of the fungal community structure, with soil communities clearly separated from those of bark and wood, consistent with previous studies [29, 30]. Nonetheless, cross-compartmental transfer cannot be ruled out. Soil, bark, and wood may act as interconnected reservoirs, with propagules dispersed via wind, rain splash, pruning wounds, or root–stem continuity. While microhabitat filters and host defenses likely constrain colonization success, several GTD-associated pathogens have been reported in multiple grapevine compartments, including roots and rhizosphere [30]. Thus, while filtering defines community composition, ecological connectivity across compartments remains possible. It should be noted that differences in multivariate dispersion contributed to the strong microhabitat effect, as soil communities were more variable than those of bark and wood. This variability is consistent with the heterogeneous nature of soil environments compared to plant tissues, and may partly explain the pronounced separation of soil samples in ordination space.

When analyzing each microhabitat separately, vintage exerted the strongest effect on fungal community composition, particularly in soil. In contrast, the importance of season, cultivar, and health state was varied depending on the microhabitat. Although these factors explained a significant share of variance, R^2^ values remained relatively low. This suggest that additional, unmeasured variables likely influence community assembly. Such residual variance is common in microbial ecology, and reflects the ecological complexity. It should also be noted that replication at the individual plant level was limited, as only a small number of vines per cultivar and health state were included. While the multiyear sampling design increases the robustness of temporal comparisons, this relatively sparse design may reduce the power to detect subtle differences among groups and could lead to underestimation of within-group variability. Consequently, some effects, particularly those related to cultivar or health state, may represent conservative estimates, and non-significant results should be interpreted with caution. In grapevines, microbial interactions, microclimatic variability, episodic dispersal and the heterogeneity of the soil may all contribute to shaping community dynamics.

In wood, health state exerted a stronger influence than cultivar, whereas a recent study from Greece [31] reported the opposite, with cultivar explaining 23% of variation and GTD status only 3.5%. In soil, annual changes accounted for ~ 15% of variation, consistent with previous reports identifying vintage as a major driver of soil fungal communities [32]. We observed pronounced interannual shifts, with both composition and functional group diversity declining in 2021, likely reflecting drier conditions. Reduced richness may have favored drought-tolerant taxa, explaining the compositional turnover. Similar interannual declines were reported for epiphytic fungi in Greece [33], where annual effects outweighed seasonal ones. The strong vintage effect is plausibly linked to climatic variability, which can alter inoculum pressure (sporulation, dispersal), host susceptibility (drought stress, wound healing), and microbial dispersal (rain splash, wind, humidity). Drought in particular exacerbates GTD symptoms by impairing host defenses, reducing xylem conductivity, and facilitating pathogen colonization [15, 34]. Although our dataset cannot disentangle these mechanisms, the observed patterns support the idea that climatic variability cascades through multiple ecological pathways to restructure grapevine mycobiomes.

The abundance of GTD pathogens was highest in plant parts, while generalist saprotrophs were most diverse in soil, consistent with the findings of a study from Spain [35]. Although the study focused solely on the plant’s endorhizosphere, they observed a lower abundance of plant pathogens and a higher abundance of saprotrophs in soil compared to plant parts. In our study, for generalist saprotrophs, mycoparasites, non-GTD wood saprotrophs, non-GTD plant pathogens, and when considering all fungi, soil was the most diverse microhabitat, suggesting a significant restriction in fungal colonization of plant organs likely due partly to environmental filtering by differences in abiotic conditions as well as to plant defense mechanisms in the case living tissues, such inner wood.

Non-GTD pathogens were equally abundant in healthy and Esca-diseased plants, whereas GTD pathogens were significantly more abundant and diverse in symptomatic vines. This contrasts with our previous study [36], where no differences were detected, likely due to the smaller sample size and single-time sampling of one cultivar (furmint) across three terroirs. A recent study from the same region [37] with multiple time points similarly reported higher GTD pathogen abundance and richness in symptomatic plants. In our dataset, differences were driven mainly by *Botryosphaeria*, *Eutypella*, and *Phaeoacremonium*, with *Botryosphaeria* reads two orders of magnitude higher in symptomatic plants. Diversity was also greater in symptomatic vines, particularly for *Botryosphaeria*, *Eutypella*, and *Fomitiporia*. These findings align with Paolinelli et al. [38], who linked pathogen abundance, rather than mere presence, to disease symptoms. Interestingly, *Phaeomoniella* was more frequent in asymptomatic plants, consistent with Bruez et al. [39] and supporting its likely endophytic nature. Esca-related fungi may thus function as latent saprobes or endophytes [40], transitioning to pathogenicity under host stress. Their dominance in woody tissues may result from both competitive exclusions, where aggressive colonizers suppress others, and habitat filtering, where the properties of decayed xylem favor a narrow pathogenic guild.

Our findings underscore the central role of microhabitat in structuring grapevine mycobiomes and the complex interplay of abiotic and biotic factors. Vintage effects, particularly drought, strongly influenced community diversity, highlighting the need to consider climatic variability in GTD management. From a plant protection perspective, strategies that integrate microbial ecology with viticultural practices and enology will be essential for sustainable grape production. Future work should further investigate how microhabitats shape fungal diversity and function, which could inform targeted interventions. The elevated abundance of GTD pathogens in symptomatic plants also points to the importance of early detection and microhabitat-specific disease management.

## Supplementary Information

Below is the link to the electronic supplementary material.

Supplementary Material 1

## Data Availability

The dataset generated during and/or analyzed during this study are available in the GenBank database (ID KIEY00000000) https://www.ncbi.nlm.nih.gov/nuccore/2872206689. A BioProject was generated (PRJNA1073309) https://www.ncbi.nlm.nih.gov/bioproject/PRJNA1073309.
