# Supplementary material for: Unveiling the mycobiome of healthy and Esca diseased grapevines: fungal community dynamics across different microhabitats, seasons, years, and cultivars
Source: Environ Microbiome. 2026 Jun 12;21:102. doi: 10.1186/s40793-026-00911-w (PMC13487917; doi:10.1186/s40793-026-00911-w)
Supplement: Supplementary file 1 [file 40793_2026_911_MOESM1_ESM.docx]

| **Factor** | **Levels** | **Replicates per level** | **Total samples** |
| --- | --- | --- | --- |
| Microhabitat | soil, bark, wood | 1 | 3 |
| Health state | asymptomatic, Esca-symptomatic | 2 | 4 |
| Year (vintage) | 2020, 2021 | 1 | 2 |
| Season | winter, summer | 1 | 2 |
| Cultivar | leányka, kékfrankos, cabernet sauvignon, chardonnay | 1 | 4 |
| **Total** | – | – | **196** |

Supplementary table 1. Experimental design showing factors, levels, replicates per level, and sample distribution for our experiment
